# The effect of isolation precautions on care processes and medical outcomes in patients colonized with MRSA

**DOI:** 10.3205/dgkh000333

**Published:** 2019-11-29

**Authors:** David Labus, Leonie Weinhold, Joerg Heller

**Affiliations:** 1 Krankenhaus Maria Hilf, Department of Internal Medicine, Bad Neuenahr, Germany; 2 University Hospital Bonn, Department of Medical Biometry, Informatics and Epidemiology, Bonn, Germany

**Keywords:** methicillin-resistant Staphylococcus aureus, patient isolation, infection control, outcome and process assessment (health care), diagnostic techniques and procedures, patient safety

## Abstract

**Background:** Isolation precautions used in methicillin-resistant *Staphylococcus aureus* (MRSA) infection control are effective in inhibiting pathogen transmission, but may cause unintended consequences in medical care. In addition, while costs attributed to MRSA are known to be substantial, little is known about their reimbursement in the German Diagnosis Related Groups (G-DRG) payment system. The aim of our study was to examine the effect of isolation precautions used in MRSA infection control on care processes, patient outcomes and deliver reliable data on MRSA-attributed reimbursement.

**Methods:** A retrospective, matched cohort study of inpatients admitted to a general care teaching hospital in Bad Neuenahr, Germany, between January 1^st^, 2016, and December 31^st^, 2017 was performed. Patients isolated for MRSA colonization were matched to non-isolated controls based on age, gender, MRSA-adjusted Patient Clinical Complexity Level (Ma-PCCL) and Major Diagnostic Category (MDC). Main outcome measures on care processes and patient outcomes included adverse events, patient complaints, 30-day readmission rates, length of stay, type of discharge, and performance of instrument-based diagnostics. MRSA-attributed reimbursement was measured by conducting two separate G-DRG groupings, one with inclusion of MRSA-related codes and one without.

**Results:** A total of 26,059 patients were admitted to Maria Hilf Hospital in Bad Neuenahr, Germany, during the study period. We identified 304 patients isolated for MRSA colonization. Compared to non-isolated matched controls, those on isolation precautions for MRSA colonization acquired about 45% more pressure ulcers and experienced significant delays in the performance of radiological diagnostics and echocardiographs. Patients isolated for MRSA colonization received about 49% fewer echocardiographs and had about 38% fewer abdominal ultrasound exams performed compared to non-isolated matched controls. A non-significant tendency towards fewer discharges to rehabilitation clinics and higher mortality rates were observed in patients isolated for MRSA colonization. Reimbursements were negligibly affected when MRSA-related codes were integrated by the grouper.

**Conclusion:** Isolation precautions are associated with adverse consequences for care processes. These consequences need to be mitigated in order to justify placing patients at risk.

## Zusammenfassung

**Hintergrund:** Isolierungsmaßnahmen sind effektiv in der Prävention der Übertragung von Methicillin-resistenten *Staphylococcus aureus* (MRSA)-Stämmen. Mögliche negative Auswirkungen solcher Isolierungsmaßnahmen auf die Versorgungsqualität wurden bislang unzureichend untersucht. Auch ist wenig über die Vergütung der Kosten für Isolierungsmaßnahmen bekannt. Ziel dieser Studie war es daher, den Einfluss von MRSA-Isolierungsmaßnahmen auf die medizinische Versorgungsqualität zu untersuchen und Daten über die MRSA-assoziierte Vergütung zu ermitteln.

**Methode:** Es wurde eine retrospektive, gematchte Kohorten-Studie mit Patienten durchgeführt, die 2016 und 2017 in einem Krankenhaus der Grundversorgung in Bad Neuenahr, Deutschland, hospitalisiert waren. MRSA-isolierte Patienten wurden mit nicht-isolierten Kontrollen, basierend auf Alter, Geschlecht, MRSA-adjusted Patient Clinical Complexity Level (Ma-PCCL) und Major Diagnostic Category (MDC) verglichen. Primäre Endpunkte dieser Studie waren Komplikationen, Patientenbeschwerden, 30-tägige Wiederaufnahmerate, Verweildauer, Art der Entlassung sowie die Ausführung apparativer Diagnostik. Die MRSA-assoziierte Vergütung wurde mittels zwei verschiedener G-DRG Kalkulationen, eine mit Integration MRSA-relevanter Codes und eine ohne, berechnet.

**Ergebnisse:** 304 MRSA-isolierte Patienten wurden mit 304 gematchten, nicht-isolierten, nicht MRSA-kolonisierten Kontrollen verglichen. Isolierte Patienten hatten 45% mehr Dekubiti. Sie wiesen eine signifikante Verzögerung in der Durchführung von radiologischen Untersuchungen und Echokardiographien auf und erhielten 49% weniger echokardiographische und 38% weniger abdomensonographische Untersuchungen als nicht isolierte gematchte Kontrollen. Bei isolierten Patienten wurde eine nicht signifikante Tendenz zu weniger Entlassungen in Rehabilitationszentren und eine höhere Mortalität nachgewiesen. Die Vergütung wurde kaum durch MRSA-relevante Codes beeinflusst.

**Schlussfolgerung:** Isolierungsmaßnahmen zur Prävention der MRSA-Übertragung sind mit einem negativen Einfluss auf die medizinische und pflegerische Versorgungsqualität assoziiert und nicht adäquat vergütet.

## Introduction

Although the prevalence of methicillin-resistant *Staphylococcus aureus* (MRSA) in hospitals has decreased in industrialized countries within recent years, MRSA infection control remains a worldwide challenge [1]. The most important risk factors for MRSA infection include MRSA colonization and proximity to others with MRSA colonization or infection [2]. National guidelines concerning MRSA infection control recommend the utilization of isolation precautions for patients hospitalized with documented or suspected colonization or infection with infectious pathogens [3]. These precautions have been demonstrated to be effective in controlling MRSA outbreaks as well as preventing transmission of the pathogen [4]. However, unintended adverse consequences due to MRSA-related isolation may occur. Previous research has found negative psychological effects [5], [6], [7], [8], [9], reduced satisfaction with care, reduced patient safety [10], [11] and higher healthcare costs related to additional expenses attribute to MRSA [12], [13], [14]. 

The aim of our study was to further investigate the sole effect of isolation precautions used in MRSA infection control on care processes and patient outcomes in addition to providing data on MRSA-attributed reimbursement costs according to the German Diagnosis Related Groups (G-DRG) payment system. To achieve this, we examined several markers, including the performance of certain instrument-based diagnostics, adverse events, patient complaints, readmission rates, and type of discharge for patients isolated for MRSA colonization, and compared them with non-isolated matched controls. In addition, we calculated MRSA-attributed reimbursement costs according to the G-DRG payment system.

## Materials and methods

We performed a retrospective, matched cohort study with patients consecutively admitted to Maria Hilf Hospital, a 300-bed general teaching hospital in Bad Neuenahr, Germany, between January 1^st^, 2016 and December 31^st^, 2017. Data were collected from hospital administrative databases, hospital administrative reports, and medical records. The study was approved by the research ethics board of the medical association Rhineland Palatinate, Germany. We identified all patients isolated for MRSA colonization. Patients with MRSA infection were excluded in order to avoid confounding of outcomes primarily associated with MRSA infection status. Patients isolated for MRSA colonization were matched to non-isolated patients by age (±5 years), gender, MRSA-adjusted Patient Clinical Complexity Level (MA-PCCL) and Major Diagnostic Category (MDC) at a ratio of 1:1. Matching was done using R Version 3.3.2 [15].

### Isolation precautions

Isolation precautions used in MRSA infection control were based on the most recent recommendations from the commission for hospital hygiene and infection control in Germany (KRINKO) [3]. All patients were screened upon admission for MRSA colonization, using nasal, rectal/groin, and wound swabs. Patients with a positive MRSA culture were directly assigned isolation precautions. Isolation precautions were designed to inhibit pathogen transmission through direct (person to person) and indirect (environmental) contact. These precautions included placing patients in private rooms, requiring visitors and personnel to wear personal protective equipment, restricting patient movement, and limiting use of a given piece of medical equipment to a single patient. In terms of patient transport, patients and involved personnel were required to wear personal protective equipment, bedsheets were changed beforehand, and contaminated surfaces were disinfected. All MRSA cases were confirmed by an infection control specialist and were documented in the hospital infection control report.

### Demographic characteristics

Demographic characteristics were obtained from hospital administrative databases, hospital administrative reports, and medical records. The hospital’s discharge database is a database that contains the demographic, administrative, and clinical data of all patients admitted to the hospital. Data on MRSA colonization status on the other hand was obtained from the hospital’s infection control report. The hospital infection control report is a database where all patients with MRSA colonization or infection are continuously documented by an infection control specialist. As MRSA colonization status was not automatically cross-referenced to the hospital’s discharge database, patients were individually allocated by a trained researcher with their case number. Patients’ comorbidities were summarized by an adjusted Patient Clinical Complexity Level without MRSA colonization (Ma-PCCL). PCCL is commonly used in Germany as a measure of patients’ comorbidities [16]. In the PCCL, results range from 0 to 6 (0=not severe; 6=severe). The adjusted PCCL (Ma-PCCL) was defined by excluding MRSA colonization from the grouping. Thus, comparison of patient outcome between MRSA-colonized and MRSA-free patients with an equal adjusted PCCL reflects the impact of MRSA colonization and subsequent isolation. Adjusted PCCLs were calculated by a trained data analyst using G-DRG grouping software (3M Suite^®^). 

Diagnoses upon admission were summarized as Major Diagnostic Categories (MDC) and were obtained from the hospital’s discharge database. MDC are used in the G-DRG to categorize diagnoses upon admission, based on the organic etiology. Data on age and gender were also derived from the hospital’s discharge database.

### Patient outcome data

Data on acquired pressure ulcers and falls were obtained from incident reports that are filled out by healthcare workers and were deposited in the electronic medical record (i.med^®^). In patients with multiple events, only one event per admission was included. Data on laboratory adverse events were obtained by carefully reviewing patients’ laboratory charts within the electronic medical record (i.med^®^). Hyper-/hyponatraemia and hyper-/hypokalaemia were defined as any deviation from the reference values, which were not present upon admission [17]. Acute kidney failure was defined according to the KDIGO Clinical Practice Guideline for Acute Kidney Injury [18]. Our reviewer was not blinded to the patient’s group allocation. By using exact definitions for reporting data, we aimed to limited review bias. 

Data on patient complaints, 30-day readmission rates, length of stay, and type of discharge were collected from the hospital’s discharge database. The 30-day readmission rate was defined as the number of patients that were rehospitalized within 30 days of discharge. Length of stay was defined as time in days from admission to discharge, transfer to another hospital or rehabilitation clinic, discharge against medical advice, or death. Type of discharge was defined as number of patients who were discharged, transferred to another hospital, transferred to a rehabilitation clinic, discharged against medical advice, or died. 

Data from echocardiographs and abdominal ultrasounds were obtained from two sources. First, registration times were individually searched by a trained researcher in the electronic medical record (i.med^®^). Second, times of examinations were obtained from the hospital’s medical diagnostic report database (Clinic WinData^®^), where all diagnostic reports and times of examinations for completed echocardiographs and abdominal ultrasound exams are collected. We only included diagnostics with complete available data, including registration times, times of examinations and medical reports. 

Data from radiological diagnostics were derived from the hospital’s radiological administrative database. The radiological administrative database is a database that contains orders and times of examinations for all radiological diagnostic tests conducted on hospitalized patients. Radiological diagnostics can be ordered for a requested time point. For our calculations, we defined the requested time as the registration time point, because this was the time the clinician determined as adequate. Diagnostics that were performed prior to the time for which they were requested were defined as “0” delay. Following consultation with the Radiology Department, performed diagnostics that were recorded ≥7 days after the requested time were excluded from our calculations, as these were thought to be documentation errors. 

Performed diagnostics were allocated to their cohort (patients isolated for MRSA colonization or non-isolated matched control). The absolute numbers of performed diagnostics were counted. Delays in diagnostics were quantified as time in hours from registration until implementation.

### MRSA-attributed reimbursement

Data on MRSA-attributed reimbursement were derived using G-DRG grouping software (3M Suite^®^). Therefore, an additional grouping was performed, in which the MRSA-related codes for MRSA colonization (ICD U 80.0 !) and MRSA infection control procedures (OPS 8-987) were excluded. Reimbursements with and without inclusion of MRSA-related codes were calculated in Euros per group. MRSA-attributed reimbursement was defined as the difference in calculated reimbursement costs in Euros between groups. Effective weight is used in the G-DRG as a variable that determines the financial complexity of a case and thereby its reimbursement. Reimbursements can be calculated by multiplying the effective weight with the base rate [19].

### Statistical analysis

Binary outcomes (adverse events, readmission rates, patient complaints, type of discharge) were summarized as absolute numbers with percentages. Continuous outcomes (length of stay, effective weight, reimbursement DRG, performance of instrument-based diagnostics) were either summarized by means with standard deviation (SD) or medians with range, when outcome variables showed skewed distribution. Group differences regarding categorical outcome variables were assessed by McNemar’s tests for paired dichotomous data. The difference in the frequencies of instrument-based diagnostic examinations between the groups was assessed by binomial tests. Given that the distribution of the variables in radiological diagnostics, echocardiography, and abdominal ultrasound (delay of instrument-based diagnostic outcome variables) was right-skewed, log-transformation was applied prior to the analysis. In order to account for multiple measurements per patient for the delay of instrument-based diagnostic outcome variables, linear mixed models were used. In addition, the group difference for length of stay was determined using the Wilcoxon signed-rank test. P-values were considered statistically significant when p≤0.05. All analyses were conducted using R Version 3.6.0 [15].

## Results

### Baseline data

**Figure 1:**
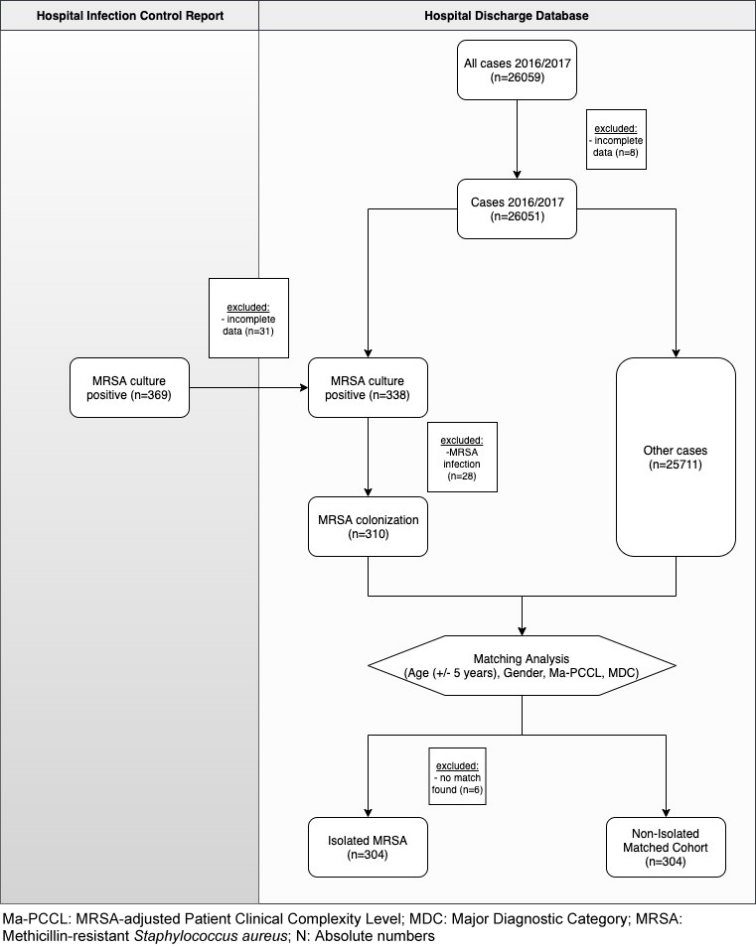
Enrolment flowchart

The dataset included all 26,059 patients who were admitted to Maria Hilf Hospital in Bad Neuenahr, Germany during the study period (Figure 1 (Fig. 1)). Eight patients were excluded due to incomplete data. 369 patients with MRSA colonization or infection were identified from the hospital’s infection control report and allocated to the hospital’s discharge database. Of these 369 patients, 31 were excluded due to incomplete data. Additionally, patients with MRSA infection were excluded (n=28), as they did not fulfill the inclusion criteria. After allocation to the hospital’s discharge database was finished, 310 patients isolated for MRSA colonization were used for the matching analysis. Six patients who met the inclusion criteria did not have a matched control and were excluded from the analysis. Finally, 304 patients isolated for MRSA colonization and 304 matched controls were identified (Figure 1 (Fig. 1)). The mean age of the group of patients isolated for MRSA colonization was 75.7 years, and 75.6 years in the control group (SD=16.78 and 16.73, respectively). In both groups, 51.3% of the patients were women. The mean PCCL of patients was 1.9 (SD=1.54, Table 1 (Tab. 1)). 

### Performance of instrument-based diagnostics

Patients isolated for MRSA colonization showed a significant delay in the performance of radiological diagnostics (median 1.03 vs. 0.97 hours; p=0.0410) and echocardiographs compared to non-isolated matched controls (Table 2 (Tab. 2)). Echocardiographs were performed an average of 20 hours later than in non-isolated matched controls (median 47.79 vs. 26.12 hours; p=0.0209). In comparison to non-isolated matched controls, patients isolated for MRSA colonization were about 49% less frequently examined with echocardiography (26 vs. 51, p=0.0059) and received about 38% fewer abdominal ultrasound exams (42 vs. 68, p=0.0167). No statistically significant differences of numbers of performed radiological diagnostics (699 vs. 693, p=0.8934) or time to abdominal ultrasounds (21.77 vs. 21.98 hours, p=0.9809) between groups were found (Table 2 (Tab. 2)).

### Patient outcome data

Patients isolated for MRSA colonization acquired about 45% more pressure ulcers than did non-isolated matched controls (12.5% vs. 6.9%; p=0.0251) during their hospitalization (Table 3 (Tab. 3)). Numbers of falls, hyper-/hyponatremias, hyper-/hypokalaemias, acute kidney failures, patient complaints, and 30-day readmission rates did not differ significantly between groups. Furthermore, length of stay did not differ significantly between groups. Regarding type of discharge, patients isolated for MRSA colonization tended to be less likely to be discharged to a rehabilitation clinic (4.3% vs. 5.9%; p=0.3827) and showed a trend towards higher mortality rates (6.6% vs. 3.9%; p=0.2159) compared to non-isolated matched controls (Table 3 (Tab. 3)).

### MRSA-attributed reimbursement according to the G-DRG

The calculated effective weight and reimbursement decreased only slightly after MRSA-related codes in the G-DRG were excluded from the grouping. MRSA-attributed reimbursement amounted to on average €320.30 in our sample (Table 4 (Tab. 4)). 

## Discussion

The present study is the first in Germany to address the effect of isolation precautions on medical outcome in patients with MRSA colonization as compared to non-isolated matched controls.

The results of our study indicate that patients isolated for MRSA colonization received fewer echocardiographs and abdominal ultrasounds, experienced delays in the performance of radiological diagnostics and echocardiographs, and acquired more pressure ulcers compared to non-isolated matched controls. Furthermore, isolation resulted in a non-significant tendency towards fewer transfers to rehabilitation clinics and increased in-hospital mortality. Compared to matched controls, isolated patients were not more likely to have a fall incident, develop hyper-/hyponatraemia, hyper-/hypokalaemia or acute kidney failure, have a longer stay, be readmitted within 30 days after discharge, or fill in a patient complaint. 

Prior work has demonstrated the efficacy of isolation precautions in inhibiting pathogen transmission and infection rates [4]. Aside from that, previous research outside Germany has already addressed negative consequences associated with isolation precautions. These studies reported negative psychological effects [5], [6], [7], [8], [9], reduced satisfaction with care, reduced patient safety [10], [11] and higher healthcare-associated costs [12], [13], [14] among isolated patients. However, negative effects on certain processes of care and patient outcomes may emerge from these procedures.

In a study by Stelfox et al., one of the largest studies on the safety of patients isolated for infection control, patients isolated for MRSA colonization or infection were more likely than non-isolated matched controls to experience supportive care failures such as pressure ulcers, falls, or electrolyte disorders [10]. Our study partially supports this finding, as isolated patients acquired about 45% more pressure ulcers than did their matched controls. In contrast, no significant differences in fall rates, hyper-/hyponatraemias, hyper-/hypokalaemias, or acute kidney failures were found. Critiques of earlier studies pointed out that adjustment for severity of underlying illness might have been inappropriate in earlier studies by including MRSA infections, as these are associated with more severe clinical outcomes than MSSA infections or colonization [20], [21]. Therefore, it is possible that our matched design adjusted better for severity of underlying illnesses by only focussing on MRSA colonized patients, thus yielding a more precise representation of the specific effect of isolation. 

Our work also contributes novel information regarding healthcare worker contact and clinical examinations. In a prospective, observational cohort study by Saint et al., physicians were about half as likely to examine patients to whom contact precautions applied [22]. Others reported more unrecorded or incompletely recorded vital signs, as well as more days without physician or nurses’ notes in patients isolated for MRSA colonization or infection, compared to non-isolated controls [10]. In addition, isolated patients with congestive heart failure received fewer diagnostic tests, medication changes, and follow-up appointments than did their non-isolated controls [10]. To our best knowledge, this is the first study to examine the impact of isolation precautions on the performance of certain instrument-based diagnostics in clinical practice. We demonstrated about 49% fewer echocardiographs performed and about 38% fewer abdominal ultrasound exams performed, as well as delays in the performance of radiological diagnostics and echocardiographs among isolated patients. It can be hypothesized that these failures were primarily related to isolation precautions, as patient transport is inhibited, and instruments and examination rooms needed to be disinfected after examinations. Clinicians might have been inclined to perform fewer diagnostics in isolated patients to avoid additional efforts and loss of time. Taken together, decreased numbers and delays in certain instrument-based diagnostics might have resulted in fewer accurate diagnoses and later initiation of necessary treatments.

Earlier studies have mainly focussed on in-hospital mortality but not on the type of discharge. We found a tendency towards fewer discharges to rehabilitation clinics in patients under isolation. A possible reason for this might be that beds for isolated patients in out-of-hospital facilities are rare, which makes it more difficult to organize such transfers. Our results are also somewhat consistent with the finding that isolated patients experience delayed transfers to long-term care facilities [23]. 

It can be hypothesized that patients who are transferred to rehabilitation clinics require more supportive care and are thereby at a higher risk of being affected by associated failures.

When no such beds are available, clinicians might be forced to discharge patients differently or patients may experience prolonged hospitalization while awaiting transfer to another facility. Further research is needed to investigate the impact of these delays on long-term patient outcome.

Apart from the demonstrated negative effects on certain care processes, an increased economic burden associated with isolation precautions has been addressed by several authors [12], [13], [14].

Most recently, a cost analysis study by Hübner et al., which was performed among isolated MRSA patients in a university hospital in Germany, calculated additional costs of €8,673.04 per case. Opportunity costs, such as blocking of beds, made up the largest part of these costs [12]. Such a cost calculation was not possible in our study population due to the retrospective character as well as missing data on hospital expenses. However, data on MRSA-attributed reimbursement according to the G-DRG payment system are scarce. In this study, we demonstrated an additional reimbursement of €320.30 per case. Compared to the estimated costs reported in earlier studies, this finding represents a wide discrepancy between additional MRSA-attributed costs and its reimbursement. Kiesel et al. reported similar results for MRSA-attributed costs in patients admitted to a regional hospital with specialized care [24]. Consistent with our findings, they reported only little additional reimbursement of €15.45 with MRSA-attributed costs of €7,732.33 per patient. Our findings indicate slightly higher MRSA-attributed reimbursement, which may be related to the recency of our study, as reimbursements are adjusted by the INEK (Institut für das Entgeldsystem in Krankenhaus gGmbH) annually and have a tendency to increase. In contrast to Kiesel et al., we integrated the G-DRG relevant code for MRSA colonization status (ICD U 80.0 !) into our calculation, potentially resulting in greater reimbursement. The negative effects demonstrated in this study mainly relate to preventable complications that may be addressed by implementation of higher healthcare-worker:patient ratios in the care of isolated patients. However, it is obvious that hospitals have no opportunity to remedy these shortcomings without an equitable reimbursement in the G-DRG payment system.

The results of our study should be interpreted in the context of the following limitations. First, this was a retrospective study. Data on adverse events were collected from the hospital administrative report files. Although healthcare workers were obliged to fill in incident reports immediately, we cannot be certain that all adverse events were reported correctly. Second, our study was restricted to patients isolated for MRSA colonization, in order to determine the sole effect of patient isolation as precisely as possible. However, we cannot rule out that patients isolated for different pathogens show different outcomes. Third, in Germany, the severity of a patient’s underlying illnesses is measured with the PCCL, which is part of the G-DRG payment system. The quality of this instrument has not been evaluated. We acknowledge that this instrument is not common in other countries, and earlier studies mostly utilized the Charlson Comorbidity Index for their matching analysis. This might result in findings that are less comparable to other studies in the literature. On the other hand, the PCCL integrates more comorbidities than the Charlson Comorbidity Index, which may give a more precise representation of the severity of underlying illnesses.

Nevertheless, the study has some important implications that are worth mentioning. Despite the beneficial effects associated with isolation precaution in infection prevention, we demonstrated that these measures are associated with negative consequences on certain care processes. The finding of certain instrument-based diagnostics being performed less often and with and longer delays is important, as this may cause less accurate diagnoses with later initiation of needed treatments. 

Our finding that reimbursement increased only slightly when MRSA-related codes were integrated by the grouper raises the question of whether infection control procedures are addressed sufficiently in the G-DRG. Therefore, in the future, studies that investigate both, MRSA-attributed costs and reimbursement, are required. 

## Conclusion

Isolation precautions have shown to be an effective tool in MRSA infection prevention. Nevertheless, these procedures can have negative consequences on care processes and medical outcome, specifically in a lower number of and longer delays in certain instrument-based diagnostics as well as more pressure ulcers acquired. In order to justify isolation precautions used in MRSA infection control, future infection control programs and hospitals should implement measures to mitigate associated risks in Germany.

## Notes

### Competing interests

The authors declare that they have no competing interests.

### Acknowledgements

We thank Heike Fassbender and Klaus Wilhelm for technical support in data acquisition, and Christopher Brown for valuable critical comments. 

## Figures and Tables

**Table 1:**
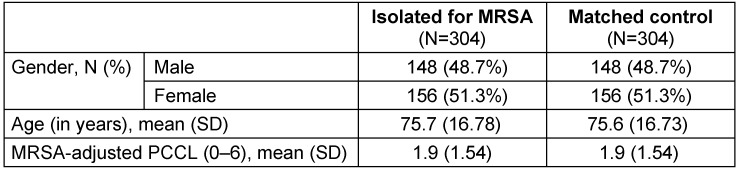
Demographic characteristics of patients isolated for MRSA colonization and non-isolated matched controls

**Table 2:**
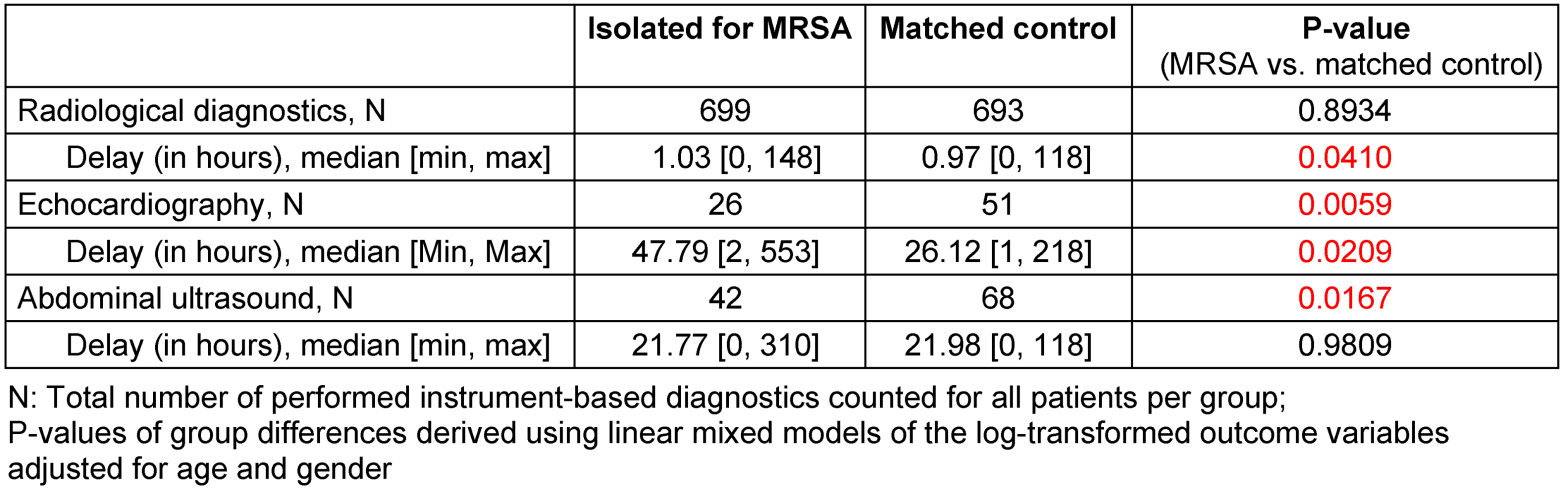
Performance of instrument-based diagnostics in patients isolated for MRSA colonization and non-isolated matched controls

**Table 3:**
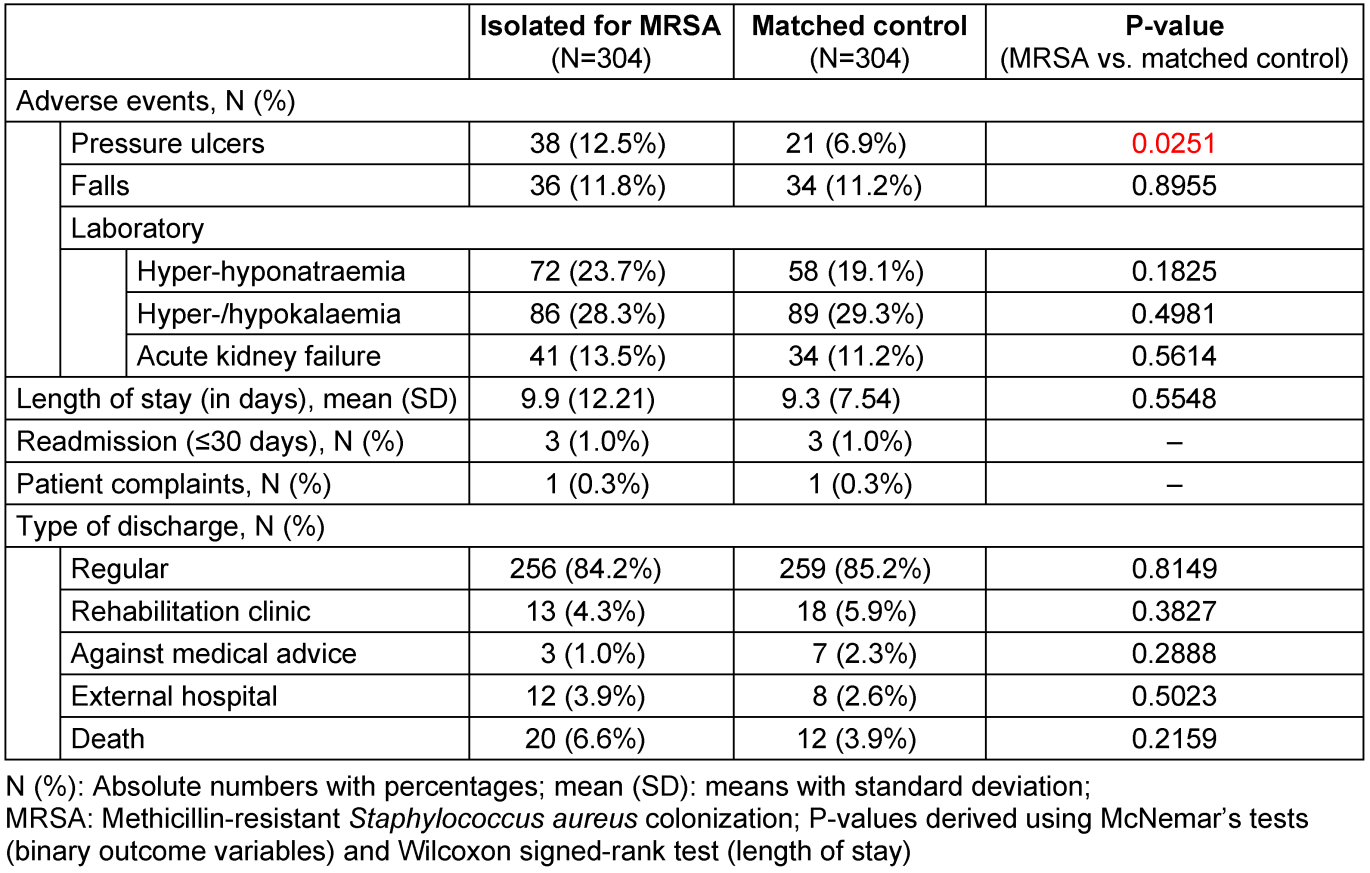
Patient outcome data in patients isolated for MRSA colonization and non-isolated matched controls

**Table 4:**
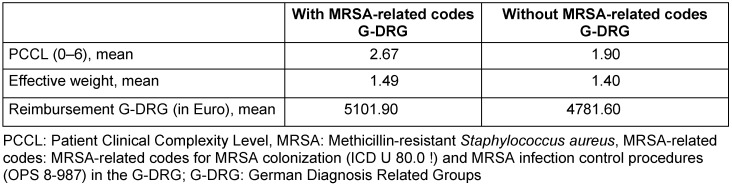
G-DRG reimbursement comparing grouping with and without MRSA-related codes
